# An Invasive Whitefly Feeding on a Virus-Infected Plant Increased Its Egg Production and Realized Fecundity

**DOI:** 10.1371/journal.pone.0011713

**Published:** 2010-07-22

**Authors:** Jian-Yang Guo, Gong-Yin Ye, Sheng-Zhang Dong, Shu-Sheng Liu

**Affiliations:** 1 Ministry of Agriculture Key Laboratory of Molecular Biology of Crop Pathogens and Insects, Institute of Insect Sciences, College of Agriculture and Biotechnology, Zhejiang University, Hangzhou, Zhejiang, China; 2 Zhejiang Provincial Key Laboratory of Biometrology and Inspection and Quarantine, College of Life Sciences, China JiLiang University, Hangzhou, Zhejiang, China; CNRS - Université Aix-Marseille, France

## Abstract

**Background:**

Plant-pathogenic begomoviruses have a complex association with their insect vectors. The interactions of begomoviruses and reproduction of their vectors are poorly understood. *Bemisia tabaci* is known to transmit many begomoviruses, and the spread of *B*. *tabaci*, especially the B and Q ‘biotypes’, has been accompanied by the epidemics of begomoviruses. One of these identified disease-causing agents was *Tomato yellow leaf curl China virus* (TYLCCNV).

**Methodology/Principal Findings:**

In this study, we compared the egg production and realized fecundity of two ‘biotypes’ or putative species of the whitefly *B*. *tabaci*, including the alien invasive B and the indigenous ZHJ1 from Zhejiang, China, feeding on either healthy or TYLCCNV-infected tobacco plants. The ovary of the whitefly was composed of 12–22 telotrophic ovarioles. According to the morphology of the oocytes and level of yolk content, oocytes in ovarioles were divided into four developmental phases (I-IV). Significantly higher proportion of immature oocytes (phase II, III) and mature oocytes (phase IV) was observed in ovary of females that fed on TYLCCNV-infected tobacco compared to that on healthy plants. Moreover, there was significant increase of eggs laid of B whitefly that fed on TYLCCNV-infected tobacco plants during the early developmental stages. In contrast, the proportion of oocytes of different developmental phases and eggs laid had no significant differences between ZHJ1 whiteflies feeding on TYLCCNV-infected and non-infected host plants.

**Conclusions/Significance:**

The invasive B whitefly benefits from feeding on a begomovirus-infected plant through increased egg production and realized fecundity.

## Introduction

Plant pathogen-vector systems are characterized by complex interactions. Some plant- pathogenic geminiviruses have caused serious problems to crops [1]–[3]. Most of these plant viruses are vectored by insects and the interactions between plant viruses and their insect vectors have been shown to be beneficial, neutral, or antagonistic, depending on the species involved [4], [5]. Generally, some of the plant viruses were observed to have intimate molecular interactions with their vectors, including movement of the viruses from the gut back to the mouthparts through salivary gland, and the transcription and replication of virus within the vector [6]. Moreover, it was reported that the longevity and fecundity of insect vectors were affected when fed on virus-infected plants [7]–[9]. However, the mechanisms underlying the interactions between the plant viruses and the reproduction of its vector insect are poorly understood.

The whitefly *Bemisia tabaci* (Hemiptera: Aleyrodidae) is genetically diverse and a vector of many begomoviruses [10]–[13]. Recent phylogenetic analysis combined with a pattern of reproductive isolation among genetic groups within *B*. *tabaci* indicates that the complex contains at least 24 cryptic species, some of which have been referred to as ‘biotypes’ in the last 20 years [14], [15]. As the separation at the species level within the *B*. *tabaci* complex is yet to be fully resolved, the commonly used term ‘biotypes’ to refer cryptic species has been sometimes retained [16].

Two cryptic species of the *B. tabaci* complex, i.e., the Mediterranean and the Middle East-Minor Asia 1 [14], which have commonly referred to as the Q biotype (Q hereafter) and the B biotype (hereafter B), have spread into much of the rest of the world from its presumed origin in the past 20 years, and have displaced some indigenous cryptic species of this species complex in the regions of invasion [2], [17], [18]. However, the mechanisms underlying the displacement of indigenous whiteflies by B are not thoroughly clear. Previously, it has been found that mating behavioral interactions between different cryptic species of the whitefly are important in the invasion by B [2], [19]. In another study, Jiu et al. [8] demonstrated that the invasive B feeding on begomovirus-infected plants substantially increased its longevity and fecundity compared to that on uninfected plants while the indigenous ZHJ1 whitefly (hereafter ZHJ1, which belongs to the Asia II 3 cryptic species as named by Dinsdale et al. [14]) was unable to do so, and speculated that this indirect vector-virus mutualism through their shared host plant may accelerate the population increase of the invasive B in the field [8]. However, the physiological mechanisms of this indirect mutualism are not known.

Moreover, compared to its performance on uninfected plants, B feeding on begomovirus-infected plants performed better [20], [21], similarly [3], [22], [23], or less well [24]. Virus-infected plants undergo changes that could affect the biology of insect vectors [25]. These variations of relationships may affect the process of biological invasion and the displacement of indigenous species by invaders when the invasive and indigenous organisms occur with niche overlap but differ in the interactions [8]. Therefore, a more thorough understanding on how the begomoviruses (as well as others) affect the reproduction of their vectors seems necessary.

The virus vectors of this study include the B and ZHJ1 whiteflies, which are known to belong to different putative species of the *B*. *tabaci* species complex [14], [15]. The notorious invasive B entered China in the late 1990s and now is the predominant or only biotype in many regions of the country [2], [26], [27]. The virus, *Tomato yellow leaf curl China virus* (TYLCCNV) is a whitefly-transmitted begomovirus that has become widespread in south China for years [28], [29]. Here, the anatomy of the reproductive organ of the whitefly was observed, the percentage of oocytes in ovaries at different developmental phases and the fecundity between the invasive B and the indigenous ZHJ1 whiteflies feeding on healthy and TYLCCNV-infected tobacco plants were compared.

## Materials and Methods

### Whiteflies

Two cryptic species of the whitefly *Bemisia tabaci* (Gennadius) species complex were used. The B whitefly (GenBank accession no. AJ867555) population was first collected from cabbage, *Brassica oleracea* var. *capitata* L. (Cruciferae), and the indigenous ZHJ1 whitefly (GenBank accession no. AJ867556) population from cotton, *Gossypium hirsutum* L., in 2003 in Hangzhou, Zhejiang, China (32.2 °N, 120.1 °E, with an elevation of 6 m a.s.l.) [3], [8]. Stock cultures of two cryptic species were maintained on cotton *G*. *hirsutum* L. cv. Zhemian 1973 in separate climate chambers at 28±1 °C, 14 h light: 10 h darkness and 70±10% r. h. The purity of the cultures was monitored every 3-5 generations using the random amplified polymorphic DNA-polymerase chain reaction (RAPD-PCR) technique [30], and measures were taken to use only pure sub-cultures of the two cryptic species for experiments. Viruliferous whitefly colonies were reared separately at the same condition as described above.

### Viruses

Infectious clones of TYLCCNV and their satellite DNA molecules (named DNA *β*) constructed previously [28], [29] were used as inocula, and the viruses were maintained on plants of tobacco *Nicotiana tabacum* L. cv. NC89.

### Plants

Tobacco *Nicotiana tabacum* L. cv. NC89, a host plant of TYLCCNV, and cotton (cv. Zhemian 1973), a non-host plant of TYLCCNV, were used. Uninfected tobacco and cotton plants were grown in insect-proof cages under natural lighting and ambient temperature in screen houses as described in Jiu et al. [8]. The virus-infected tobacco plants were acquired by inoculating with TYLCCNV and its DNA *β* at the 4–5 true-leaf stage by agro-inoculated as previously described [1], [28], [29]. Healthy and TYLCCNV-infected tobacco plants were grown to the 6–7 true-leaf stage for experiments. The virus infection of test plants was judged by the characteristic symptoms caused by the virus and further confirmed by molecular marks as previously described [8]. All plants were watered every 3–4 days as necessary and fertilized once a week. All experiments were conducted at 26±1 °C, 40–60% relative humidity, and a photoperiod of 14 h light: 10 h darkness.

### Light microscopy

The whitefly adults of different developmental stages reared on cotton plant were insufflated into centrifuge tube on ice. The ovaries of each female were then dissected in phosphate buffered saline (PBS) (pH 7.4) and the epidermis was removed. Different types of ovarioles were separated using a stretched microcapillary. The morphology of ovary samples and ovarioles were photographed with a phase contrast microscope (Leica 216, Germany) fitted with a light source. Digital images were captured using a digital camera and modified using Adobe Photoshop®.

### Development of ovaries in initially non-viruliferous adults feeding on healthy or virus-infected tobacco

These experiments were conducted to examine the realized fecundity of adult whiteflies which were reared on cotton from egg to pupa, and then transferred upon emergence onto healthy or virus-infected tobacco plants. The experiment was carried out concurrently using the procedure as described earlier by Jiu et al. [8] with a few modifications. Approximately 600 pairs of newly emerged (0–12 h) adult whiteflies were collected from the culture on cotton, and divided randomly into two groups, 300 pairs of adults each. Each group was used for inoculation onto either healthy or TYLCCNV-infected tobacco plants. On each type of plants, 10 females were dissected everyday for counting the number of oocytes at different developmental phases in ovaries until 15 d after eclosion.

### Fecundity of initially non-viruliferous adults feeding on healthy or virus-infected tobacco

The number of eggs laid on healthy or virus-infected tobacco by the whiteflies was examined using the clip-cages described by Zang et al. [30]. Each cage contained one male and one female. The total number of eggs laid was examined daily for the first 8 d after eclosion using the microscope (Leica, Germany) as leaves covered by the clip-cages started to wilt after 8 d.

### Statistical analysis

All statistical analysis were conducted using the DPS^©^ package (Version 8.01 for Windows) [31]. To analyze the effects of TYLCCNV infection on the reproduction of the same cryptic species of whiteflies feeding on healthy and virus-infected plants, data were subjected to Student *t*-test. The effects of virus infection and age on the reproduction of B or ZHJ1 were analyzed using two-factors ANOVA and Tukey's multiple range test (MRT). The total number of oocytes and eggs laid of different cryptic species of whiteflies feeding on healthy and virus-infected plants in the first 2 d were analyzed using two-factors ANOVA and Tukey's MRT. All proportion data were transformed by arcsine root before analysis, but untransformed means are presented.

## Results

### Anatomy of ovary in the whiteflies

**Figure 1:**
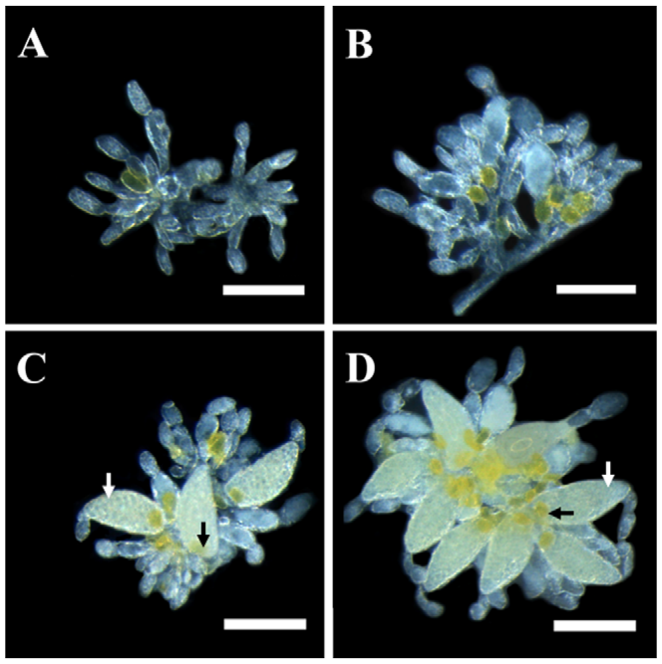
Ovary of B whitefly at different developmental stages after eclosion. A: the ovary of freshly emerged whitefly; B: 1–2 d after eclosion; C: 3–10 d after eclosion; D: 11–14 d after eclosion. Mature oocytes were showed with white arrows. Bacteriocyte sphere was showed with blank arrows. Scale bar: 0.10 mm.

**Figure 2:**
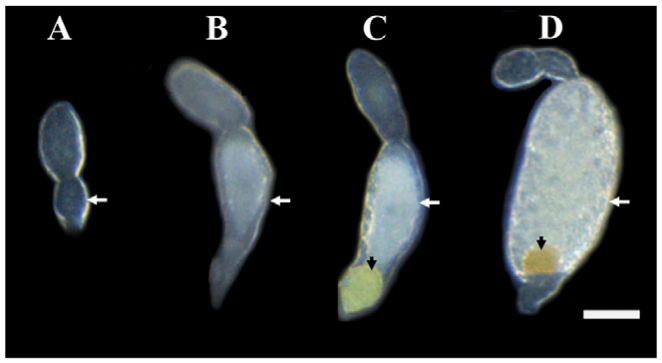
Oocytes of different developmental phases in the ovarioles of whitefly. A, B, C and D are termed as phase I, phase II, phase III and phase IV, respectively (showed with white arrows). Bacteriocyte sphere was showed with blank arrows. Scale bar: 0.05 mm.

The ovaries of the whiteflies are formed by a mass of ovarioles, of which the number for a pair of ovary is ranged from 12–22 in various developmental stages. Every ovariole contains two or three oocytes in various stages of maturity (Fig. 1). The ovary can be divided into four stages during its development: (1) stage 1, within 2 h after eclosion, wherein one or more oocytes were transparent (Fig. 1A); (2) stage 2, 1–2 d after eclosion, wherein one or more oocytes were opaque and with yolk contents (Fig. 1B). The bacteriocytes formed around the immature oocytes and some of them were transferred into the immature eggs in the region where the pedicel was ultimately formed; (3) stage 3, 3–10 d after eclosion, wherein one or more oocytes were filled with yolk contents and an increasing number of ovarioles and mature oocytes were observed (Fig. 1C); (4) stage 4, 11–14 d after eclosion, wherein most oocytes were full with yolk content. Mature oocytes were always observed at the third stage (Fig. 1C) and the number of mature oocytes increased to the maximum from 7 to 11 d after eclosion (Fig. 1D).

According to the main morphological characteristics of oocytes and the level of yolk content, the oocytes were classified into four developmental phases, separately named phase I, II, III and IV (Fig. 2). No yolk protein was observed in phase I oocyte. The phase II and III were identified by the presence of yolk content. Oocytes with<50% yolk content were named phase II and those with >50% yolk content phase III. The phase ІV were mature eggs. A rounded bacteriocyte sphere was transferred into oocyte at the terminal of ovariole in phase III and embedded in mature oocyte (Fig. 2).

### Effects of TYLCCNV on proportions of oocytes in ovaries of whiteflies

**Figure 3:**
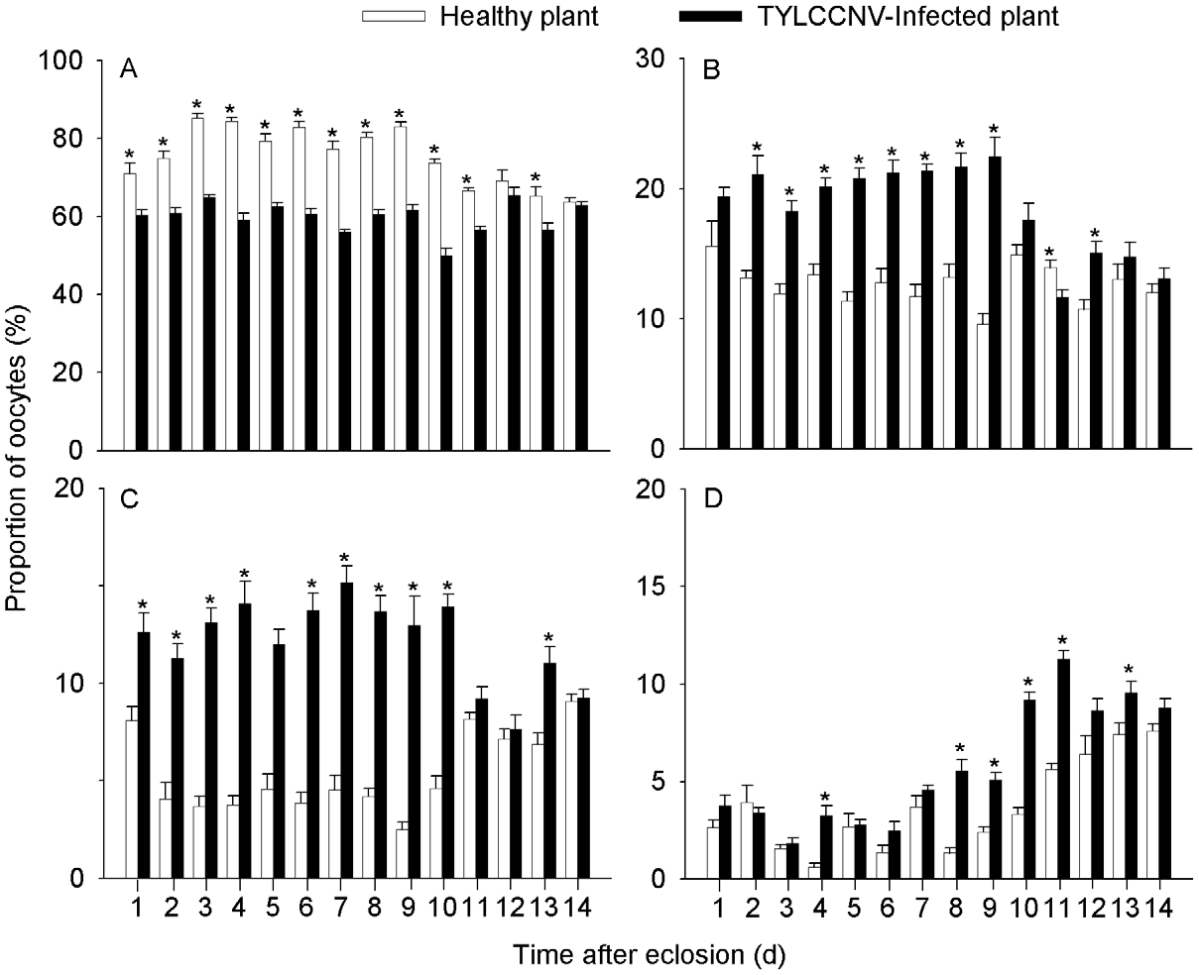
Proportion of different phase oocytes in the ovary of B whitefly. A, B, C, D: phase I, II, III, IV. The data are expressed as means ± SE (n = 10). At the same day, values followed by the asterisk (*) represent significant differences (*P*<0.05, Student *t*-test).

**Figure 4:**
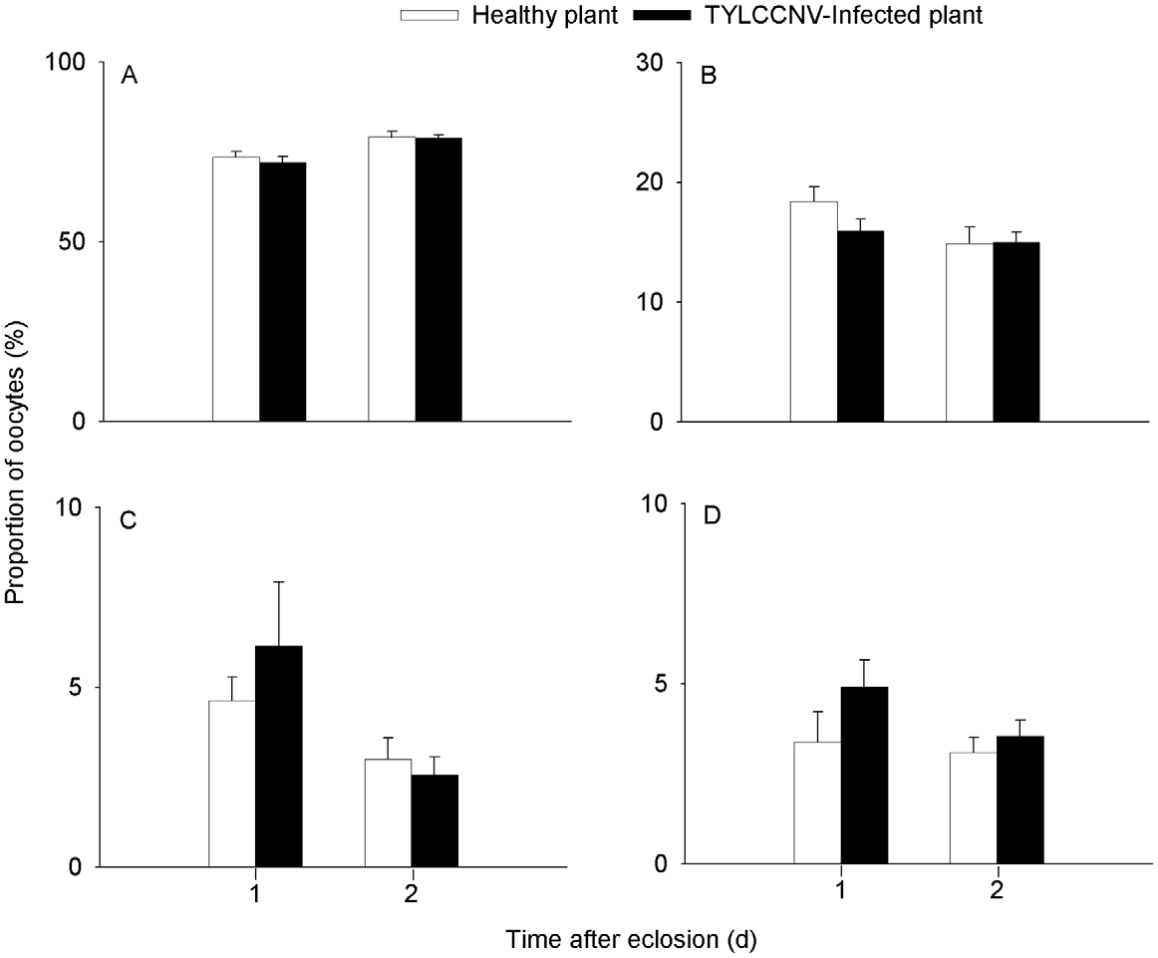
Proportion of different phase oocytes in the ovary of ZHJ1 whitefly. A, B, C, D: phase I, II, III, IV. The data are expressed as means ± SE (n = 10).

**Figure 5:**
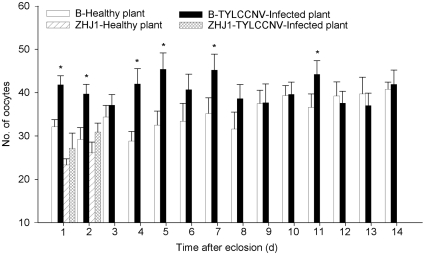
Total numbers of oocytes in the ovaries of two cryptic *B*. *tabaci*. The data are expressed as means ± SE (n = 10). At the same day, values followed by the asterisk (*) represent significant differences (*P*<0.05, Student *t*-test).

B adults feeding on virus-infected plants had a significantly lower proportion of phase I oocytes but significant higher proportions of phase II, III, and IV oocytes, compared to the B adults feeding on healthy plants (Table 1; Figure 3). In contrast, the proportions of oocytes at different phases in ZHJ1 were similar between adults feeding on healthy plants and those feeding on virus-infected plants (Table 1; Figure 4). In the B whiteflies, compared with adults feeding on healthy plants, those feeding on virus-infected plants had a significantly higher total number of oocytes. However, in ZHJ1 no significant difference was observed between adults feeding on virus-infected and healthy plants (Fig. 5).

**Table 1:** Two-factors ANOVA on different phase of oocytes in the ovary of B and ZHJ1 whiteflies.

Parameters measured		Percentage (%) of oocytes in pairs of ovary		Two-factors ANOVA
		TYLCCNV- Infected plants	Healthy plants	
B	Phase I	59.89±6.50 b	75.51±9.66 a	*F*_A_ = 57.6880, *df* = 1; *P*<0.0001
				*F*_B_ = 1.3930, *df* = 13; *P* = 0.2793
				*F*_A×B_ = 7.7570, *df* = 13; *P*<0.0001
	Phase II	18.48±5.01 a	12.67±4.14 b	*F*_A_ = 151.3340, *df* = 1; *P*<0.0001
				*F*_B_ = 4.1150, *df* = 13; *P*<0.0001
				*F*_ A×B_ = 5.3880, *df* = 13; *P*<0.0001
	Phase III	12.12±3.90 a	4.71±0.353 b	*F*_A_ = 426.4280, *df* = 1; *P*<0.0001
				*F*_B_ = 1.9950, *df* = 13; *P* = 0.0216
				*F*_A×B_ = 11.4570, *df* = 13; *P*<0.0001
	Phase IV	9.51±0.717 a	7.11±0.603 b	*F*_A_ = 4.3230, *df* = 1; *P*<0.0480
				*F*_B_ = 5.7900, *df* = 13; *P* = 0.0017
				*F*_ A×B_ = 5.9440, *df* = 13; *P*<0.0001
ZHJ1	Phase I	75.42±6.45 a	76.32±6.66 a	*F*_A_ = 1.5310, *df* = 1; *P* = 0.4328
				*F*_B_ = 71.8390, *df* = 1; *P* = 0.0748
				*F*_ A×B_ = 0.1530, *df* = 1; *P* = 0.6979
	Phase II	15.49±3.42 a	16.64±5.31 a	*F*_A_ = 0.7850, *df* = 1; *P* = 0.5384
				*F*_B_ = 2.9790, *df* = 1; *P* = 0.3343
				*F*_A×B_ = 0.8680, *df* = 1; *P* = 0.3577
	Phase III	4.86±0.343 a	3.81±0.254 a	*F*_A_ = 0.4910, *df* = 1; *P* = 0.6108
				*F*_B_ = 4.3780, *df* = 1; *P* = 0.2838
				*F*_A×B_ = 3.5060, *df* = 1; *P* = 0.0693
	Phase IV	4.24±0.246 a	3.23±0.258 a	*F*_A_ = 3.5570, *df* = 1; *P* = 0.3104
				*F*_B_ = 2.4300, *df* = 1; *P* = 0.3631
				*F*_A×B_ = 0.4400, *df* = 1; *P* = 0.5112

Note: the value for each parameter measured is from the analysis of all data during the whole experiment period, and is expressed as mean ± standard error (n = 140). In the same row, the data followed by the same lowercase letter do not differ significantly according to two-factors ANOVA. Factor A: types of tobacco plants for feeding the *Bemisia tabaci* (TYLCCNV-infected plants versus healthy plants); and Factor B: time after eclosion.

Due to the short lifespan of ZHJ1 on healthy and virus-infected plants, these parameters were compared statistically at the first two days after emergence with B. The total number of oocytes in B was significantly higher than that of ZHJ1 (Table 2).

**Table 2:** Two-factors ANOVA on fecundity of B and ZHJ1 whiteflies feeding on healthy or TYLCCNV-infected plants in the first two days after eclosion.

Parameters	TYLCCNV-Infected plants		Healthy plants	
	B	ZHJ1	B	ZHJ1
No. of oocytes per ovary a	40.75±2.33 a	29.05±0.89 bc	30.65±1.32 b	24.7±0.82 c
No. of eggs per cage b	7.9±1.43 a	4.4±0.62 c	5.75±1.18 b	3.8±0.51 c

a Two-factors ANOVA: *F*_biotype_ = 35.8, *df* = 1; *P*<0.05; *F*_plant_ = 24.7, *df* = 1; *P*<0.05; *F*_biotype* plant_ = 3.8, *df* = 1; *P*<0.05.

b Two-factors ANOVA: *F*_ biotype_ = 77.9, *df* = 1; *P*<0.05; *F*_ plant_ = 40.5, *df* = 1; *P*<0.05; *F*_ biotype* plant_ = 60.8, *df* = 1; *P*<0.05.

In the same row, means followed by the same lowercase letters do not differ significantly according to two-factors ANOVA and Tukey's multiple-range test. Data are represented as means ± standard error (n = 20).

### Effects of TYLCCNV on fecundity of whiteflies

**Figure 6:**
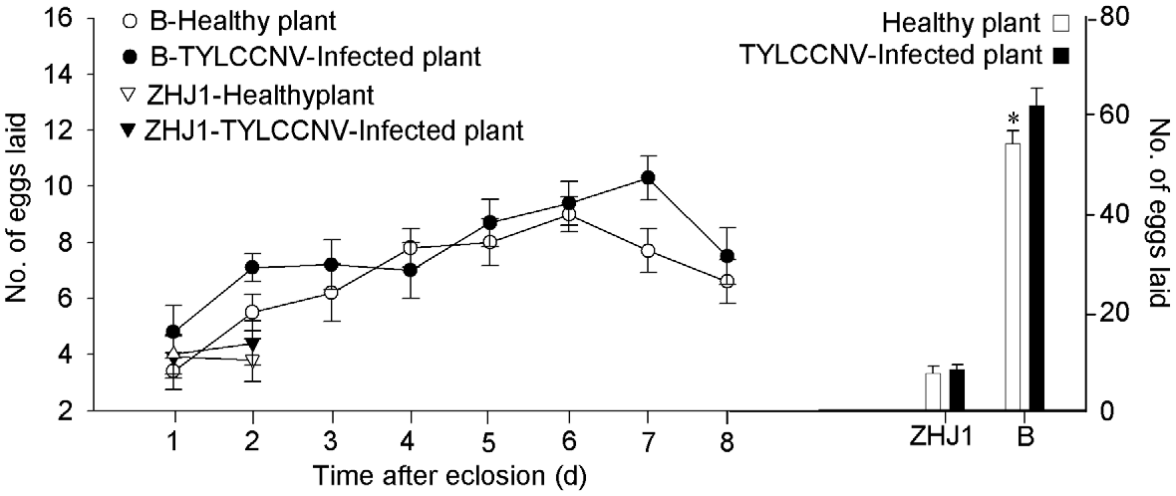
Fecundity of non-viruliferous adults fed on healthy or virus-infected tobacco of two cryptic whiteflies in the first 8 d after eclosion. The data are expressed as means ± SE (n = 10). Values followed by the asterisk (*) represent significant differences (*P*<0.05, Student *t*-test).

Compared with the ZHJ1, B whiteflies laid 5–8 times more eggs on either healthy or virus-infected plants (Fig. 6, Table 2). The B adults feeding on TYLCCNV-infected plants laid significantly more eggs than those feeding on healthy plants; in contrast, the ZHJ1 adults feeding on healthy and virus-infected plants laid similar numbers of eggs (Figure 6; Table 2)

**Figure 7:**
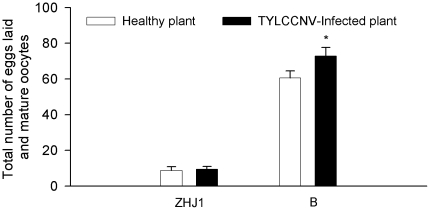
Total number of eggs plus mature oocytes of non-viruliferous adult fed on healthy or virus-infected tobacco of two cryptic whiteflies in the first 2 d after eclosion. The data are expressed as means ± SE (n = 80). Values followed by the asterisk (*) represent significant differences (*P*<0.05, Student *t*-test).

When the number of mature oocytes in ovaries and that of eggs laid was added, the total number in B adults was 4–6 time higher than that in ZHJ1 adults feeding on either healthy or virus-infected plants (Fig. 7).

## Discussion

The ovariole of insects is usually divided into three types: the polytrophic type, telotrophic type and the panoistic type [32]. In the present study, it was assumed that the ovarioles of *B*. *tabaci* belongs to the telotrophic type. This type ovariole had been reported in three species of aphid, *Stomaphis quercus*, *Phylloxera coccinea* and *Phylloxera glabra* [33], [34]. In *B*. *tabaci*, the ovaries were characterized by an increasing number of ovarioles and mature oocytes after eclosion and 12–22 ovarioles were observed in its adult stage. Our results are consistent with Gameel et al. [35] who depicted 15 ovarioles in *B*. *tabaci*. Previous reports had provided only a brief description of the ovarian structure and oogenesis in green house whitefly *Trialeurodes vaporariorum* [36]. In another report, the paired ovaries of whitefly *Aleurochiton aceris* are composed of 5 short telotrophic ovarioles [37]. In *B*. *tabaci*, each ovariole contained two or three oocytes and a germarium. Once a mature oocyte was present in ovariole, three oocytes would be observed in the same ovariole. This ovarian system, based on morphological examination of oocyte of the four developmental phases, was used to evaluate the effects of virus infection of the plants on the whitefly reproduction.

A rounded bacteriocyte sphere was observed to be transferred into the oocyte at the terminal of ovariole in oocyte III and embedded in mature oocyte. The bacteriocytes housing symbiotic bacteria had been examined in whiteflies [37]–[39]. However, the interactions of this bacteriocyte with the oogenesis and reproduction of its host were poorly known. Gottlieb et al. [38] demonstrated the presence of five types of secondary symbionts, including *Hamiltonella*, *Arsenophonus*, *Cardinium*, *Wolbachia*, and *Rickettsia*, in a bacteriocyte of whitefly. This phenomenon may be a result of the direct enclosure of the bacteriocyte in the egg during oogenesis, providing a useful mechanism for efficient vertical transmission of endosymbiotic microorganisms. These endosymbiotic microorganisms had been reported not only in whitefly, but also in aphids and other insects [40]. In the aphid *S*. *quercus*, trophocyte cytoplasm is filled with endosymbiotic microorganisms [35].

Both B and ZHJ1 whiteflies are effective vectors of TYLCCNV [41]. In the present study, compared with its fecundity on healthy tobacco, B whitefly experienced a significant increase in its mean number of oocytes in the ovary through feeding on plants infected with TYLCCNV. In contrast, the potential fecundity of ZHJ1 whitefly feeding on virus-infected plants was similar to that of adults feeding on healthy plants. Previous studies have demonstrated that B and ZHJ1 are reproductively isolated [30], and B has a wider host range than ZHJ1 and generally performs better on their shared host plants [42]. Furthermore, the tobacco cultivar used in this study is a relatively poor host plant for both whitefly cryptic species [8]. However, the invasive B has a significant advantage via its mutualism with the viruses to increase the suitability of host plants and to have a higher number of immature (phase II, III) and mature oocytes in the ovaries, while the indigenous ZHJ1 is unable to do so (Fig. 3, 4). It was assumed that the vector-virus mutualism increased the differentiation of oocytes in B during oogenesis. This was verified by the higher total number of oocytes in ovaries of B whiteflies feeding on virus-infected plants (Fig. 5). Moreover, previous studies had shown that tomato yellow leaf curl virus (TYLCV) appeared to reduce whitefly fitness, whereas tomato mottle virus (ToMoV) did not [43], [44]. It had been speculated that this may be the result of the ability of TYLCV to replicate in the whitefly. A subsequent study indicated that TYLCV transcripts were stimulated to accumulate in the whitefly [45], whereas ToMoV transcripts were not. Whether TYLCCNV could replicate and accumulate transcripts in the whitefly or not warrants investigations in the future.

In plant-pathogen-vector systems such as the one examined in this study, the pathogen depends on the arthropod herbivore vector for transmission and dispersal. Thus higher proportion of mature oocytes in the ovaries and consequently higher numbers of eggs will help increase vector population and this will in turn facilitate the spread of virus disease pandemic in the field. This kind of vector-virus mutualism in assisting in the spread of virus disease pandemic had been seen in other vector-virus-plant systems, such as some aphid-transmitted viruses in wheat, barley and oats [46], . Laboratory and field evidence indicates that this kind of vector-virus mutualism had played a significant role in driving the spread of a cassava mosaic disease pandemic in Uganda [4], [48]. Since the effect of plant viruses on the vector can vary from positive to negative, effort has been made to reveal patterns of the effects according to the type of plant-virus-vector relationships [3], [8]. However, it is not yet clear how much role the differential effects of virus-infection of plants on the two cryptic species of the whitefly have played in their interactions and competitions in the field. Our results showed that TYLCCNV infection of a host plant would help to induce different appearance of ovarian development between two cryptic species of *B. tabaci*. The yolk protein is the main composition of egg content in many insects during oogenesis. In the ovaries of the two cryptic species of the whitefly, whether the different proportions of mature oocytes were due to the synthesis and uptake of yolk protein needs to be elucidated. Further, whether feeding on the virus-infected plants could lead to structural changes of the ovaries or changes in hormone synthesis in the vectors remain to be investigated.
